# Treatment Outcomes of Sofosbuvir/Velpatasvir/Voxilaprevir in Direct-Acting Antiviral-Experienced Hepatitis C Virus Patients: A Systematic Review and Meta-Analysis

**DOI:** 10.3390/v15071489

**Published:** 2023-06-30

**Authors:** Pooja Devan, Kai Le Ashley Tiong, Jean Ee Neo, Babu P. Mohan, Karn Wijarnpreecha, Yew Chong Steve Tam, Nicola Coppola, Carmen Monica Preda, Yu Jun Wong

**Affiliations:** 1 Yong Loo Lin School of Medicine, National University of Singapore, Singapore 119077, Singapore; devan.pooja01@gmail.com (P.D.); kaileashley.tiong@gmail.com (K.L.A.T.); neojeanee@gmail.com (J.E.N.); 2 Division of Gastroenterology & Hepatology, University of Utah Health, Salt Lake City, UT 84112, USA; dr.babu.pm@gmail.com; 3 Division of Gastroenterology and Hepatology, Department of Medicine, University of Arizona College of Medicine, Phoenix, AZ 85004, USA; dr.karn.wi@gmail.com; 4 Education Resource Centre, Medical Board, Singapore General Hospital, Singapore 169608, Singapore; steve.tam.y.c@sgh.com.sg; 5 Department of Mental Health and Preventive Medicine, University of Campania “L. Vanvitelli”, 81100 Napoli, Italy; nicola.coppola@unicampania.it; 6 Clinical Institute of Fundeni, Gastroenterology and Hepatology, 022328 Bucharest, Romania; carmenmonica.preda@gmail.com; 7 Department of Gastroenterology & Hepatology, Changi General Hospital, Singapore 529889, Singapore; 8 Duke-NUS Academic Medicine Programme, SingHealth, Singapore 169608, Singapore

**Keywords:** direct-acting antiviral, treatment, ribavirin, sustained virological response, adverse effects, HCV genotype, liver cirrhosis, RAS mutation

## Abstract

About 5% of chronic hepatitis C (CHC) patients experienced treatment failure with direct-acting antiviral (DAA) treatment. The global data on the practice and treatment outcomes of Sofosbuvir/Velpatasvir/Voxilaprevir (SOF/VEL/VOX) in DAA-experienced CHC patients remains sparse. We performed a systematic review and meta-analysis to evaluate the efficacy and safety of SOF/VEL/VOX as a salvage treatment in DAA-experienced CHC patients. We searched five electronic databases from inception to 31 January 2023. The study outcomes were SVR12 and treatment-related adverse effects, with subgroup analysis performed based on genotype, cirrhosis, HCC, prior SOF/VEL exposure, and region. We identified and analyzed data from 24 studies (2877 DAA-experienced CHC patients); 17.2% had prior SOF/VEL exposure, 25% received ribavirin with SOF/VEL/VOX, and 42% had pre-treatment resistance-associated substitution (RAS) testing performed. Eastern Mediterranean had a higher pooled SVR12 than the America and Europe regions (*p* < 0.05). Predictors of SOF/VEL/VOX failure were genotype 3, active HCC, baseline cirrhosis, and prior SOF/VEL. Baseline RAS mutation and ribavirin supplementation were not associated with higher SVR12. Treatment discontinuation because of drug-related adverse events was uncommon (10 studies, 0.2%). In summary, SOF/VEL/VOX is efficacious and safe for retreatment in DAA-experienced CHC patients, even with RAS mutation. Our findings support SOF/VEL/VOX as a first-line rescue treatment for DAA-experienced CHC patients.

## 1. Introduction

Chronic hepatitis C (CHC) infection affects 56.8 million people globally, with about 1.5 million new infections every year, resulting in 400,000 deaths each year [1]. The World Health organization (WHO) has set a goal to reduce new HCV infections by 90% and death by 65% by 2030 [1]. While the global incidence of CHC has reduced from 71 million to 56.8 million, only 11 countries are currently on track to meet the WHO’s 2030 elimination target [2]. Virological cure significantly reduces liver-related complications and improves survival in CHC patients [3]. Sofosbuvir-based DAA has shown to achieved high SVR12 in real-world settings, even among HCV patients with genotype 3 [4,5]. Despite that the introduction of highly effective direct-acting antiviral drugs (DAAs) has revolutionized the CHC treatment, up to 5% of CHC patients still failed to achieve a sustained virological response at week 12 (SVR12) using DAA [6]. 

Treatment options remain limited in DAA-experienced CHC patients who fail to achieve SVR12. Retreatment of CHC patients with DAA failure could be challenging because of the emergence of resistance-associated substitution (RAS). Current guidelines recommend Sofosbuvir/Velpatasvir/Voxilaprevir (SOF/VEL/VOX) as a salvage treatment for DAA-experienced CHC patients who failed to achieve SVR12 [7]. SOF/VEL/VOX is a once-daily, oral-administering CHC treatment consisting of NS3, NS5A, and NS5B-inhibitor (Sofosbuvir 400 mg, Velpatasvir 100 mg, and Voxilaprevir 100 mg) with pangenotypic potency. The POLARIS I and 4 phase III trial reported a high treatment response among DAA-experienced CHC patients receiving SOF/VEL/VOX, with SVR12 ranging between 95% and 100% [8]. 

The global data on practice and treatment outcomes of SOF/VEL/VOX remained lacking, especially in regions such as Asia and Africa. Although some studies reported a lower SVR12 among CHC patients with liver cirrhosis or genotype 3, which is prevalent in south Asia [9,10], such findings were not consistently reported [11]. Furthermore, the predictors of treatment failure and the role of ribavirin in CHC patients receiving SOF/VEL/VOX remains unclear. Real-world data are important to determine the efficacy and safety of SOF/VEL/VOX among DAA-experienced CHC patients who were ineligible for clinical trials. To address these gaps, we performed a systematic review and meta-analysis to determine the efficacy and safety of SOF/VEL/VOX as a salvage treatment among DAA-experienced CHC patients. We also aim to determine the predictors of treatment failures following SOF/VEL/VOX among DAA-experienced CHC patients. 

## 2. Methods

### 2.1. Eligibility and Search Strategy

We followed the Preferred Reporting Items for Systematic Reviews and Meta-Analyses (PRISMA) guideline for data extraction and reporting [12]. With the help of a medical librarian, we identified all potential literature through a comprehensive search of five electronic databases (PubMed, EMBASE, Cochrane Library, ClinicalTrials.gov, and Web of Science) from inception up to 31 January 2023. The search keywords included a combination of “hepatitis C”, “Sofosbuvir”, “Velpatasvir”, “Voxilaprevir”, and “virological response” (Supplementary Table S1). We also searched the references of all included studies for potential eligible studies.

### 2.2. Study Selection

We included all studies that met one of the following inclusion criteria, regardless of publication dates and publication status. Our inclusion criteria were as follows: (1) DAA-experienced, adult CHC patients (age > 18 years old) treated with 12 weeks of SOF/VEL/VOX, with or without Ribavirin, and (2) reported data on either SVR12 or adverse events. Our exclusion criteria were (1) paediatric CHC patients; (2) animal studies; (3) studies that did not report study outcomes; (4) case reports or case series with less than 10 patients; (5) reviews, editorials, or guidelines; and (6) clinical trials. Three authors (P.D., K.L.A.T., and J.E.N.) independently performed the initial screening of the title and abstract identified in the primary search for eligibility. Any discrepancy in the article selection was resolved by discussion and consensus with the senior author (W.Y.J.). 

### 2.3. Data Extraction and Quality Assessment

The data from each study were independently extracted using a standardized form. We contacted the corresponding author to verify any missing information. The data extracted included demographics of the study participants (age and genotype of hepatitis C virus), location, sample size, RAS testing at baseline, SVR12, concurrent use of ribavirin (RBV), and treatment-related adverse events requiring discontinuation of treatment.

### 2.4. Outcomes Assessed

The outcomes studied were as follows: (1) SVR12 (both the intention-to-treat (ITT) and per-protocol (PP) analysis) and (2) frequency of adverse events leading to treatment discontinuation. The ITT analysis included all subjects treated with SOV/VEL/VOX, while the PP analysis only included SOF/VEL/VOX-treated CHC patients with available SVR12 results. Subgroup analysis was performed based on region, HCV genotype, baseline cirrhosis status, prior SOF/VEL exposure, hepatocellular carcinoma, RAS mutation, and concurrent use of ribavirin. 

### 2.5. Data Synthesis and Analysis

We used Review Manager Software version 5.3 (The Nordic Cochrane Centre, The Cochrane Collaboration, 2014) to perform our meta-analysis. The effect measures were estimated using the odds ratio (OR) for categorical outcomes and mean difference (MD) for continuous outcomes together with the respective 95% confidence interval (95%CI). The meta-analysis was performed using the random-effects model. A *p*-value of less than 0.05 was considered to be statistically significant. Study heterogeneity was assessed by the *I^2^*% statistics. 

### 2.6. Quality Assessment

The quality of all included studies was independently assessed by three authors using the Risk Of Bias In Non-Randomized Studies of Interventions (ROBINS-I) tool (non-randomized studies) [13] and the Cochrane risk-of-bias tool for randomised trials (RoB 2) (randomised studies) [14] as low risk of bias, some concerns of bias, or high risk of bias. 

## 3. Results

### 3.1. Search Results 

**Figure 1:**
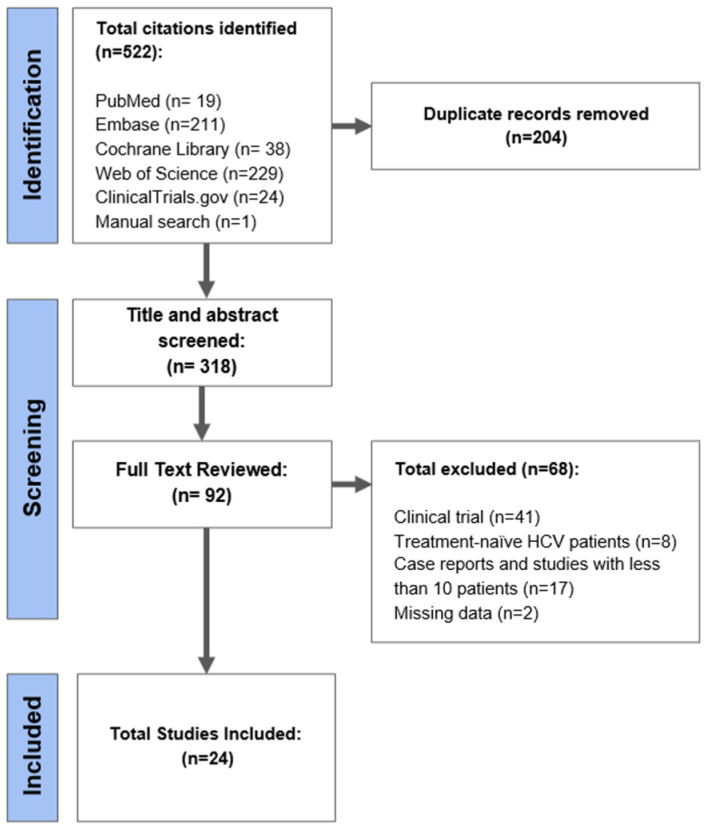
PRISMA flowchart.

From an initial total of 522 citations identified using our search strategy, 92 full texts were reviewed. We excluded 68 citations for the following reasons: clinical trial (n = 41), treatment-naïve CHC patients (n = 8), case report or study with less than 10 patients (n = 17), and missing data (n = 2). Finally, a total of 24 studies were included for analysis (Figure 1)**.** Most studies had either a low risk [6,9,15,16,17,18,19,20,21,22,23] or moderate risk of bias [10,11,24,25,26,27,28,29,30,31,32,33] (Supplementary Table S2). Most studies were reported from the European region (37.5%), followed by the Americas region (29.2%), Western Pacific region (20.8%), Eastern Mediterranean region (8.3%), and African region (4.2%). 

### 3.2. Population Characteristics

A total of 2887 DAA-experienced CHC patients from 24 studies were included. All patients received 12 weeks of SOF/VEL/VOX, with or without ribavirin. The patient characteristics of the included studies are summarised in Table 1. The cohort was predominantly male (81.0%) with a diverse HCV genotype distribution (GT1 61.4%, GT2 8.2%, GT3 21.5%, GT4 7.0%, GT6 1.2%, and indeterminate GT 0.7%). At baseline, 42% had liver cirrhosis, 17.2% had prior SOF/VEL exposure, 24.8% received ribavirin in addition to SOF/VEL/VOX, and 50% had pre-treatment RAS testing performed. There was no significant publication bias detected for all key outcomes (Supplementary Figure S1).

### 3.3. Sustained Virological Response at 12 Weeks (SVR12)

The overall SVR12 for DAA-experienced CHC patients treated with SOF/VEL/VOX based on PP and ITT analysis was 95% (95%CI: 94.0–95.8%) and 88% (95%CI: 86.7–89.5%), respectively. The SVR12 in the Eastern Mediterranean region (two studies: 98.2% (95%Cl: 96.0–99.3%)) was significantly higher than the region of Americas (seven studies: 93.2% (95%Cl; 91.4–94.7%), *p* = 0.0006), Western Pacific region (five studies: 94.7% (95%Cl: 91.3–97.1%), *p* = 0.019), and Europe region (nine studies: 95.8% (95%Cl: 94.5–96.7%), *p* = 0.04) (Supplementary Figure S2). The higher SVR in the Eastern Mediterranean region is likely due to a lower proportion of CHC patients with liver cirrhosis (6.4% vs. 35.1%, *p* < 0.001) and GT3 infection (0% vs 24.4%, *p* < 0.001). The real-world studies had a lower pooled SVR12 than the clinical trials (87.7% (95%CI: 86.4–89.2%) vs. 98.2% (95%CI: 95.9–99.2%), *p* < 0.0001). 

#### 3.3.1. HCV Genotype

**Figure 2:**
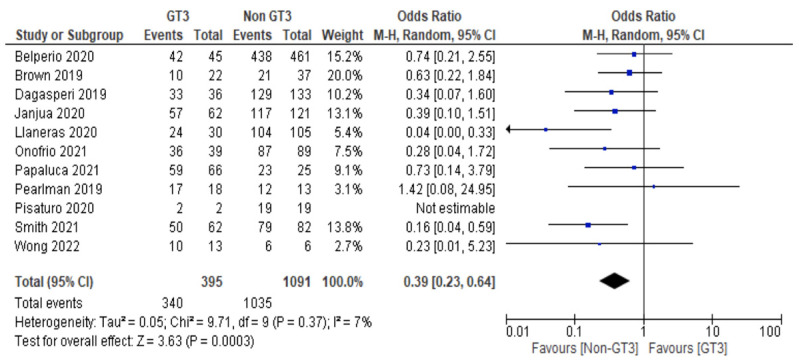
The proportion of patients with non-GT3 HCV infection versus GT3 HCV infection achieving SVR12 with SOF/VEL/VOX.

The overall SVR12 among genotype 3 (GT3) and non-GT3 DAA-experienced CHC patients treated with SOF/VEL/VOX was 86.1% (95%CI: 82.3–89.3%) and 94.9% (95%CI: 93.4–96.1%), respectively. GT3 HCV infection was associated with a lower odds of SVR12 (10 studies, OR = 0.39, 95% CI 0.23–0.64, *I*^2^ = 7%) (Figure 2).

#### 3.3.2. Liver Cirrhosis

**Figure 3:**
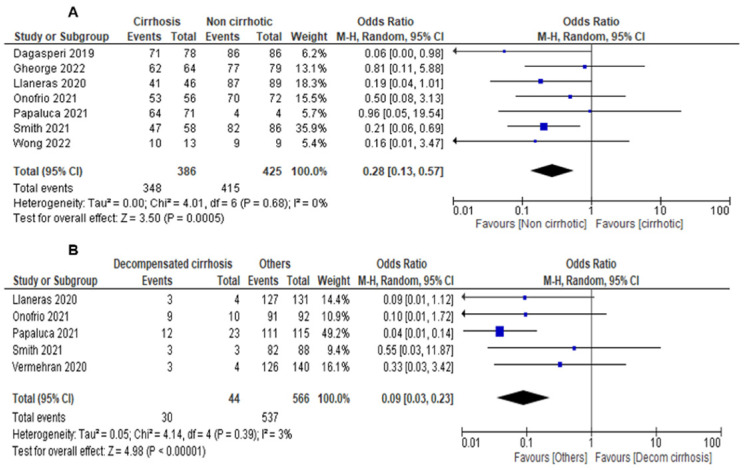
The proportion of HCV infection patients achieving SVR12 with SOF/VEL/VOX between (**A**) cirrhotic versus non-cirrhotic HCV infection and (**B**) decompensated cirrhosis versus cirrhotic HCV patients.

The overall SVR12 among cirrhotic and non-cirrhotic DAA-experienced CHC patients treated with SOF/VEL/VOX was 88.8% (95%CI: 84.9–92.1%) and 97.7% (95%CI: 95.5–99.0%), respectively. Liver cirrhosis was associated with a lower odds of SVR12 (seven studies, OR = 0.28, 95% CI 0.13–0.57, *I*^2^ = 0%) (Figure 3A). Decompensated HCV cirrhosis patients receiving SOF/VEL/VOX were associated with a lower odds of SVR12 than compensated cirrhosis patients (five studies, OR = 0.09, 95%CI: 0.03–0.23, *I*^2^ = 3%) (Figure 3B).

#### 3.3.3. Prior Sofosbuvir/Velpatasvir Exposure 

**Figure 4:**
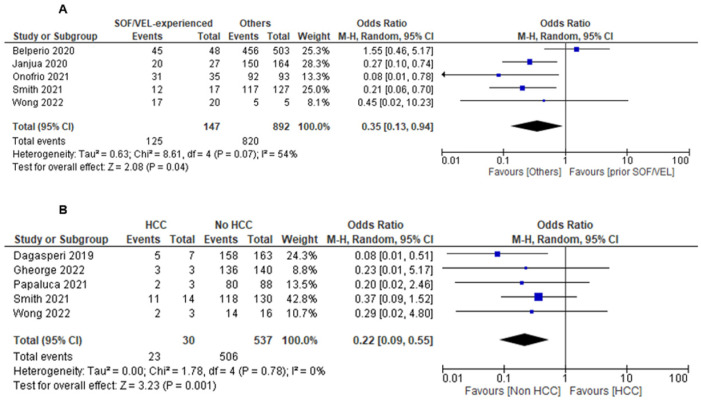
The proportion of HCV infection patients achieving SVR12 with SOF/VEL/VOX between (**A**) SOF/VEL-experienced versus SOF/VEL-naïve and (**B**) active hepatocellular carcinoma (HCC) versus no HCC.

The overall SVR12 among patients with and without prior SOF/VEL exposure while treated with SOF/VEL/VOX was 84.1% (95%CI: 78.4–89.9%) and 91.7% (95%CI: 90.0–93.5%), respectively. Prior SOF/VEL exposure was associated with a lower odds of SVR12 (five studies, OR = 0.35, 95% CI: 0.13–0.94, *I*^2^ = 54%) (Figure 4A). 

#### 3.3.4. Hepatocellular Carcinoma

The overall SVR12 among patients with and without active HCC was 71.1% (95%CI: 55.3–86.8%) and 92.8% (95%CI: 90.3–95.3%), respectively. Active HCC during SOF/VEL treatment was associated with a lower odds of SVR12 (five studies, OR = 0.22, 95% CI: 0.09–0.55, *I*^2^ = 0%) (Figure 4B).

#### 3.3.5. RAS Mutation

The overall SVR12 among patients with and without RAS mutation was 93.8% (95%CI: 89.5–98.0%) and 91.6% (95%CI: 85.1–98.1%), respectively. The odds of SVR12 were similar among DAA-experienced CHC patients receiving SOF/VEL/VOX, with or without baseline RAS testing (two studies, OR = 0.73, 95% CI 0.11–4.77, *I*^2^ = 34%) (Supplementary Figure S3).

#### 3.3.6. Ribavirin

The overall SVR12 among CHC patients receiving SOF/VEL/VOX, with or without ribavirin, was 95.2% (95%CI: 86.7–98.3%) and 96.2% (95%CI: 94.0–98.5%), respectively. The odds of SVR12 were similar among DAA-experienced CHC patients receiving SOF/VEL/VOX, with or without ribavirin (three studies, OR = 0.76, 95% CI 0.12–4.68, *I*^2^ = 55%) (Supplementary Figure S4).

### 3.4. Adverse Events

From a total of 1283 DAA-experienced CHC patients received SOF/VEL/VOX, the pooled risk of severe adverse events that occurred during SOF/VEL/VOX treatment was 1.94% (95%CI: 1.2–2.8%). The types of serious adverse events are summarised in Supplementary Table S3. Treatment-related adverse events in patients with decompensated cirrhosis were only reported in one study, where three patients developed treatment-related SAE, namely abdominal pain (n = 2) and acute kidney injury (n = 1). 

## 4. Discussion

In this meta-analysis, we found that SOF/VEL/VOX is an efficacious and safe salvage therapy among DAA-experienced CHC patients. The pooled SVR12 was high and treatment discontinuation due to treatment-related SAE was uncommon. Predictors for a lower SVR12 rate by SOF/VEL/VOX were genotype 3, active HCC, baseline cirrhosis, decompensated cirrhosis, and prior SOF/VEL exposure. Meanwhile, the presence of baseline RAS mutation and addition of ribavirin to SOF/VEL/VOX was not associated with higher SVR12. The higher overall SVR12 among the Eastern Mediterranean studies is likely attributed to a lower proportion of patients with GT3 infection and liver cirrhosis. 

SOF/VEL/VOX, being a protease-inhibitor based DAA, was discouraged in patients with decompensated cirrhosis because of potential toxicity from delayed drug clearance. Among 34 decompensated CHC patients treated with SOF/VEL/VOX in five different studies [9,30,31,32,33], none experienced treatment-related adverse events requiring early treatment cessation. SAE among decompensated cirrhosis patients was reported only in one study [29]. Meanwhile, hepatic decompensation had also been reported among compensated cirrhosis patients receiving SOF/VEL/VOX. In a large multinational registry of advanced CHC cirrhosis patients (up to CTP-class B or MELD score or 15) receiving DAA, the risk of liver decompensation was not significantly different between protease inhibitor-based versus non-protease inhibitor-based DAA [35]. Until further data are available among decompensated cirrhosis patients with CTP-class C or MELD score beyond 15, liver transplant should be considered as a definitive treatment in these patients, with protease-inhibitor-based DAA reserved for transplant-ineligible patients with close monitoring. 

Current EASL guidelines recommend the use of RBV with SOF/VEL/VOX in patients with a higher risk of treatment failure; however, the benefits of routine addition of RBV remain unclear [36]. We found that RBV did not significantly improve SVR12 in DAA-experienced patients. Given the potential side effects, such as causing anemia and jaundice, it should not be used routinely as there were no substantial benefits noted in this meta-analysis. Future studies are needed to evaluate the benefit of RBV among “difficult-to-treat” CHC patients during retreatment with SOF/VEL/VOX. 

This meta-analysis provides a comprehensive global perspective on the effectiveness and safety of SOF/VEL/VOX among DAA-experienced CHC patients. Prior to this, information on practice and treatment outcomes of SOF/VEL/VOX use among Asian and African population was limited. We acknowledge that there are limitations when interpreting our findings. Most studies did not report the compliance and potential drug interaction among patients receiving SOF/VEL/VOX. The number of patients within subgroups of decompensated cirrhosis was small because the current guidelines discourage the use of SOF/VEL/VOX in these patients. Nevertheless, the results of this meta-analysis provide important data to support the recommendation of using SOF/VEL/VOX as a first-line option among DAA-experienced CHC patients and could be considered level 1 evidence. 

To conclude, SOF/VEL/VOX is a well-tolerated and highly effective rescue therapy among DAA-experienced CHC patients who failed to achieve SVR12. Predictors of treatment failure include GT3, liver cirrhosis, and active HCC. Safety data of SOF/VEL/VOX among decompensated cirrhosis patients should be investigated in further studies. 

## Figures and Tables

**Table 1:** Summary of included studies for systematic review and meta-analysis.

No	Authors, Years	Study Design	Country	Sample Size (n)	Genotype n, (%)	Prior SOF/VEL (n/n)	Use with RBV (n)	RAS Testing at Baseline (%)	SVR12 (PP)	Cirrhosis (%)	Serious AE
1	Liu CH, 2023 [6]	Prospective, multicenter	Taiwan	107	GT1a: 1.9/GT1b: 37.4/GT2: 36.4/GT3: 6.5/GT6: 16.8/GT intermediate: 0.9	22/107	1/107	64/99	104/104	0	2/104
2	Chen S, 2022 [15]	Multicenter cohort	China	13	GT3b: 46.2/GT6: 38.4/GT1b: 15.4	10/13	0/13	NA	13/13	0	0
3	YJ Wong, 2022 [16]	Retrospective, multicenter	Singapore, Taiwan, Hong Kong, Malaysia	25	G1: 8/G2: 8/G3: 56/G6: 8/Indeterminate: 20	20/25	0/25	NA	22/25	16/25	0
4	Heo, J, 2022 [17]	Prospective, multicenter trial	Korea	33	G1b: 97/GT2: 3	0/33	0/33	33/33	33/33	9/33	1/33
5	Gheorghe L, 2022 [18]	Retrospective, multicenter	Romania	143	G1b: 100	0/143	0/143	NA	141/143	0	1/143
6	EL Kassas M, 2023 [34]	RCT, multicenter	Egypt	315	NA	0/281	140/281	NA	276/298	104/281	1/281
7	Gupta N, 2022 [19]	Prospective, multicenter trial	Rwanda	40	GT3: 2.5/G4: 95/Unknown: 2.5	0/40	0/40	33/40	39/40	NA	4/40
8	Shousha HI, 2022 [20]	Prospective, multicenter	Egypt	45	G4: 100	0/45	0/45	NA	44/45	21/45	0
9	Brown, 2019 [21]	Retrospective, single center	USA	22	GT1: 81.8/GT3: 9.1/GT4: 9.1	0/22	0/22	NA	22/22	NA	3/22
10	Hezode,2019 [24]	Multicenter cohort	France	46	GT1: 32.6/GT2: 8.7/GT3: 39.1/GT4: 17.4/GT5: 2.2	0/46	10/46	34/39	42/44	41/46	3/44
11	Janjua, 2020 [25]	Multicenter cohort	Canada	191	GT1: 54.5/GT2: 8.9/GT3: 32.5/others: 4.2	27/191	38/191	NA	182/191	NA	0
12	Belperio, 2019 [26]	Retrospective, multicenter	USA	573	GT1: 85.5/GT2: 10.5/GT3: 26.7/GT4: 6.3	49/573	0/573	0	501/551	198/573	0
13	Llaneras, 2019 [9]	Prospective, multicenter	Spain	137	GT1: 59.9/GT2: 5.1/GT3: 21.9/GT4: 10.2/Others: 2.9	8/137	0/137	43/49	128/135	46/137	0
14	Degasperi, 2019 [10]	Retrospective, multicenter	Italy	179	GT1: 57.5/GT2: 10/GT3: 23.5/GT4: 8.9	36/179	39/179	94/115	162/169	78/179	11/179
15	Pearlman, 2019 [11]	Prospective, multicenter	USA	31	GT1: 41.9/GT3: 58.1	0/31	0/31	28/31	29/31	18/31	0
16	Salazar, 2020 [27]	Prospective, multicenter	Germany, Italy, Spain	56	GT1: 36.7/GT2: 22.2/GT3: 40/GT4: 1.1	0/56	9/56	53/85	45/46	5/46	0
17	Pisaturo, 2020 [28]	Prospective, single center	Italy	21	GT1: 90.5/GT3: 9.5	17/61	0/21	19/21	21/21	6/21	0
18	Vermehren, 2020 [29]	Prospective, multicenter	Germany	110	GT1: 64.5/GT3: 30.9/GT4: 4.5	18/110	4/110	NA	100/102	30/110	6/110
19	Da, 2021 [30]	Retrospective, single-center	USA	18	GT1: 77.7/GT2: 11.1/GT3: 11.1	4/18	4/18	8/14	18/18	6/18	0
20	Onofrio, 2021 [31]	Prospective, multicenter	Canada	128	GT1: 60.2/GT2: 3.1/GT3: 30.5/GT4: 4.7/GT6: 10.8/mixed: 0.8	35/128	26/128	28/51	123/128	56/128	0
21	Papaluca, 2021 [32]	Retrospective, multicenter	Australian	97	GT1: 23.7/GT3: 72.2/GT4: 1/GT6: 3	19/97	3/97	49/54	82/91	76/97	3/97
22	Smith, 2021 [33]	Prospective, multicenter	England	144	GT1: 45.8/GT2: 2.1/GT3: 43/GT4: 6.9/GT6: 2	17/144	0/144	101/144	129/144	58/144	0
23	Graf C, 2022 [22]	Prospective, multicenter	Germany, Austria, Switzerland, Belgium	416	GT1: 53.8/GT2: 1.9/GT3: 38.9/GT4: 6	0/416	0/416	16/416	401/416	NA	0
24	Ruane, 2019 [23]	Open-label trial	USA	31	GT1: 61.3/GT2: 6.5/GT3: 25.8/GT4: 3.2/GT5: 3.2	31/31	0/31	31/31	31/31	15/31	0

Abbreviations: GT: genotype, SOF/VEL: Sofosbuvir/Velpatasvir, RBV: Ribavirin, RAS: resistance-associated substitutions, SVR12, AE: adverse events, NA: not applicable; RCT: randomised controlled trials.

## Data Availability

Data will be made available upon reasonable request.

## Supplementary Materials

The following supporting information can be downloaded at: https://www.mdpi.com/article/10.3390/v15071489/s1, Supplementary Table S1. Literature Search Strategy; Supplementary Table S2. Bias assessment using the Robins-1 tool & Cochrane Risk of Bias; Supplementary Table S3. Serious adverse effects after SOF/VEL/VOX treatment; Supplementary Table S4. PRISMA Checklist; Supplementary Figure S1. Funnel plots based on various key outcomes: (A) HCV genotype. (B) Liver cirrhosis, (C) De-compensated liver cirrhosis, (D) Sofosbuvir/Velpatasvir-experienced HCV patients, and (E) Active hepatocellular carcinoma; Supplementary Figure S2. SVR12 data based on WHO region classification; Supplementary Figure S3. The overall SVR12 rate of patients who had RAS mutation versus those with no RAS mutation; Supplementary Figure S4. The overall SVR12 rate of patients treated with Ribavirin versus without Ribavirin.
