# Supplementary material for: Treatment Outcomes of Sofosbuvir/Velpatasvir/Voxilaprevir in Direct-Acting Antiviral-Experienced Hepatitis C Virus Patients: A Systematic Review and Meta-Analysis
Source: Viruses. 2023 Jun 30;15(7):1489. doi: 10.3390/v15071489 (PMC10384139; doi:10.3390/v15071489)

SUPPLEMENTARY MATERIALS

Supplementary Table S1. Literature Search Strategy

Supplementary Table S2. Bias assessment using the Robins-1 tool & Cochrane Risk of Bias

Supplementary Table S3. Serious adverse effects after SOF/VEL/VOX treatment

Supplementary Table S4. PRISMA Checklist

Supplementary Figure S1. Funnel plots based on various key outcomes: A) HCV genotype. B) Liver cirrhosis, C) Decompensated liver cirrhosis, D) Sofosbuvir/ Velpatasvir-experienced HCV patients, and E) Active hepatocellular carcinoma

Supplementary Figure S2. SVR12 data based on WHO region classification

Supplementary Figure S3. The overall SVR12 rate of patients who had RAS mutation versus those with no RAS mutation.

Supplementary Figure S4. The overall SVR12 rate of patients treated with Ribavirin versus without Ribavirin

Supplementary Table S1: Literature Search Strategy

Search No.	Search Strategy (PubMed)
#1	(hepatitis C) OR ("hepatitis C") OR (hepatitis-C) OR HCV [All Fields]
#2	sofosbuvir [All Fields]
#3	velpatasvir [All Fields]
#4	voxilaprevir [All Fields]
#5	"SOF/VEL/VOX" [All Fields]
#6	"sofosbuvir/velpatasvir/voxilaprevir" [All Fields]
#7	#2 OR #3 OR #4 OR #5 OR #6
#8	#1 AND #7
#9	#8 [Filters: English, from 2000/1/1 - 2023/1/1]

Search No.	Search Strategy (EMBASE)
#1	'hepatitis AND c' OR 'hepatitis c' OR 'hepatitis-c' OR hcv
#2	'sofosbuvir'/exp OR sofosbuvir
#3	velpatasvir
#4	'voxilaprevir'
#5	'sofosbuvir/velpatasvir/voxilaprevir'
#6	'sof/vel/vox'
#7	#2 OR #3 OR #4 OR #5 OR #6
#8	#1 AND #7
#9	#9 AND [english]/lim AND [01-01-2000]/sd NOT [02-01-2023]/sd

Search No.	Search Strategy (CENTRAL)
#1	TITLE-ABS-KEY hepatitis c OR "hepatitis C" OR "hepatitis-C" OR HCV
#2	TITLE-ABS-KEY sofosbuvir
#3	TITLE-ABS-KEY velpatasvir
#4	TITLE-ABS-KEY voxilaprevir
#5	TITLE-ABS-KEY "sofosbuvir/velpatasvir/voxilaprevir"
#6	TITLE-ABS-KEY "SOF/VEL/VOX"
#7	#2 OR #3 OR #4 OR #5 OR #6
#8	#1 AND #7
#9	#8 with Cochrane Library Publication date from Jan 2000 to Jan2023

Search No.	Search Strategy (Web of Science)
**#1**	(((ALL=(hepatitis c)) OR ("hepatitis C")) OR ("hepatitis-C")) OR (HCV)
**#2**	ALL=sofosbuvir
**#3**	ALL=velpatasvir
**#4**	ALL= voxilaprevir
**#5**	ALL="sofosbuvir/velpatasvir/voxilaprevir"
**#6**	ALL="SOF/VEL/VOX"
**#7**	#2 OR #3 OR #4 OR #5 OR #6
**#8**	#1 AND #7
**#9**	#8, Dates from 2000-01-01 to 2023-01-01

Search No.	Search Strategy (Clinical Trials)
**Condition or disease**	(((hepatitis C) OR ("hepatitis C")) OR ("hepatitis-C")) OR (HCV)
**Other Terms**	((((sofosbuvir) OR (velpatasvir)) OR (voxilaprevir)) OR (SOF/VEL/VOX)) OR (sofosbuvir/velpatasvir/voxilaprevir)

Supplementary Table S2: Bias assessment using the Robins-1 tool & Cochrane Risk of Bias

Study	Confounding	Selection Bias	Classification of Intervention	Deviation from Intervention	Missing Data	Measurement of reported results	Overall risk of bias
Liu C, 2023 [6]	Low Risk	Low Risk	Low Risk	Low Risk	Moderate Risk	Low Risk	Low Risk
Chen S, 2020 [15]	Low Risk	Low Risk	Low Risk	Low Risk	Low Risk	Low Risk	Low Risk
Wong YJ, 2022 [16]	Low risk	Low Risk	Low Risk	Low Risk	Low Risk	Low Risk	Low Risk
Jeong H, 2022 [17]	Low Risk	Low Risk	Moderate Risk	Low Risk	Moderate Risk	Low Risk	Low Risk
Brown P, 2019 [21]	Low Risk	Low Risk	Low Risk	Low Risk	Low Risk	Low Risk	Low Risk
Hezode C, 2019 [24]	Low Risk	Low Risk	Moderate Risk	Moderate Risk	Low Risk	Low Risk	Moderate Risk
Janjua N, 2020 [25]	Low Risk	Moderate Risk	Moderate Risk	Moderate Risk	Low Risk	Low Risk	Moderate Risk
Belperio PS, 2019 [26]	Moderate Risk	Moderate Risk	Low Risk	Low Risk	Moderate Risk	Low Risk	Moderate Risk
Llaneras J, 2019 [9]	Low Risk	Low Risk	Low Risk	Low Risk	Low Risk	Low Risk	Low Risk
Degasperi E, 2019 [10]	Low Risk	Low Risk	Moderate Risk	Moderate Risk	Moderate Risk	Low Risk	Moderate Risk
Pearlman B, 2019 [11]	Moderate Risk	Moderate Risk	Low Risk	Low Risk	Low Risk	Low Risk	Moderate Risk
Salazar AD, 2020 [27]	Low Risk	Moderate Risk	Moderate Risk	Moderate Risk	Moderate Risk	Low Risk	Moderate Risk
Pisaturo M, 2020 [28]	Moderate Risk	Moderate Risk	Low Risk	Low Risk	Moderate Risk	Low Risk	Moderate Risk
Vermehren J, 2020 [29]	Low Risk	Low Risk	Moderate Risk	Moderate Risk	Low Risk	Low Risk	Moderate Risk
Da BL, 2021 [30]	Low Risk	Low Risk	Moderate Risk	Moderate Risk	Low Risk	Low Risk	Moderate Risk
Onofrio FQ, 2021 [31]	Low Risk	Low Risk	Moderate Risk	Moderate Risk	Low Risk	Low Risk	Moderate Risk
Papaluca T, 2021 [32]	Low Risk	Low Risk	Moderate Risk	Moderate Risk	Low Risk	Low Risk	Moderate Risk
Smith DA, 2021 [33]	Low Risk	Moderate Risk	Low Risk	Low Risk	Low Risk	Low Risk	Moderate Risk
Gheorghe L, 2022 [18]	Moderate Risk	Low Risk	Low Risk	Low Risk	Low Risk	Low Risk	Low Risk
Gupta N, 2022 [19]	Low Risk	Low Risk	Low Risk	Low Risk	Low Risk	Low Risk	Low Risk
Ruane P, 2019 [23]	Low Risk	Low Risk	Low Risk	Low Risk	Low Risk	Low Risk	Low Risk
Shousha H, 2022	Low Risk	Low Risk	Low Risk	Moderate	Low Risk	Low Risk	Low Risk

[20]				Risk			
Graf C, 2022 [22]	Moderate Risk	Low Risk	Low Risk	Low Risk	Moderate Risk	Low Risk	Low Risk
**Cochrane Risk of Bias**	**Selection Bias**	**Performance Bias**	**Selection Bias**	**Detection Bias**	**Attrition Bias**	**Reporting Bias**	**Other Biases**
EL Kassas, 2023 [34]	Unclear	Unclear	Unclear	Unclear	Low risk	Low risk	Nil

Supplementary Table S3: Serious adverse effects after SOF/VEL/VOX treatment

**Serious Adverse Events**	**Frequency**	**Drug related?**
De novo HCC	10	6 Unrelated, 2 unrelated, 2 Unclear
Hepatic Decompensation	5	3 Unrelated, 2 Unclear
Acute Kidney Injury	4	3 Unclear, 1 related
Death*	3	2 Unrelated, 1 Unclear
Recurrent HCC	2	Unrelated
Severe Nausea and Vomiting	1	Unclear
Anemia	1	Unclear
Diabetes	1	Unclear
GI Bleeding	1	Unclear
Vaginal Cancer	1	Unclear
Urothelial Carcinoma	1	Unclear
Abdominal Hernia	1	Unclear
Cholecystectomy	1	Unclear
Acute-on-chronic liver failure	1	Unclear
Hip fracture	1	Unrelated
Humerus fracture	1	Unrelated

*No mortality directly attributed to SOF/VEL/VOX

Supplementary Table S4: PRISMA Checklist

Section and Topic	Item #	Checklist item	Page
**TITLE**			
Title	1	Identify the report as a systematic review.	1
**ABSTRACT**			
Abstract	2	See the PRISMA 2020 for Abstracts checklist.	3
**INTRODUCTION**			
Rationale	3	Describe the rationale for the review in the context of existing knowledge.	4
Objectives	4	Provide an explicit statement of the objective(s) or question(s) the review addresses.	4
**METHODS**			
Eligibility criteria	5	Specify the inclusion and exclusion criteria for the review and how studies were grouped for the syntheses.	5
Information sources	6	Specify all databases, registers, websites, organisations, reference lists and other sources searched or consulted to identify studies. Specify the date when each source was last searched or consulted.	5
Search strategy	7	Present the full search strategies for all databases, registers and websites, including any filters and limits used.	5
Selection process	8	Specify the methods used to decide whether a study met the inclusion criteria of the review, including how many reviewers screened each record and each report retrieved, whether they worked independently, and if applicable, details of automation tools used in the process.	5
Data collection process	9	Specify the methods used to collect data from reports, including how many reviewers collected data from each report, whether they worked independently, any processes for obtaining or confirming data from study investigators, and if applicable, details of automation tools used in the process.	5
Data items	10a	List and define all outcomes for which data were sought. Specify whether all results that were compatible with each outcome domain in each study were sought (e.g. for all measures, time points, analyses), and if not, the methods used to decide which results to collect.	5
	10b	List and define all other variables for which data were sought (e.g. participant and intervention characteristics, funding sources). Describe any assumptions made about any missing or unclear information.	5
Study risk of bias assessment	11	Specify the methods used to assess risk of bias in the included studies, including details of the tool(s) used, how many reviewers assessed each study and whether they worked independently, and if applicable, details of automation tools used in the process.	5
Effect measures	12	Specify for each outcome the effect measure(s) (e.g. risk ratio, mean difference) used in the synthesis or presentation of results.	
Synthesis methods	13a	Describe the processes used to decide which studies were eligible for each synthesis (e.g. tabulating the study intervention characteristics and comparing against the planned groups for each synthesis (item #5)).	6
	13b	Describe any methods required to prepare the data for presentation or synthesis, such as handling of missing summary statistics, or data conversions.	6
	13c	Describe any methods used to tabulate or visually display results of individual studies and syntheses.	6
	13d	Describe any methods used to synthesize results and provide a rationale for the choice(s). If meta-analysis was performed, describe the model(s), method(s) to identify the presence and extent of statistical heterogeneity, and software package(s) used.	6

	13e	Describe any methods used to explore possible causes of heterogeneity among study results (e.g. subgroup analysis, meta-regression).	6
	13f	Describe any sensitivity analyses conducted to assess robustness of the synthesized results.	6
Reporting bias assessment	14	Describe any methods used to assess risk of bias due to missing results in a synthesis (arising from reporting biases).	6
Certainty assessment	15	Describe any methods used to assess certainty (or confidence) in the body of evidence for an outcome.	6
**RESULTS**			
Study selection	16a	Describe the results of the search and selection process, from the number of records identified in the search to the number of studies included in the review, ideally using a flow diagram.	6
	16b	Cite studies that might appear to meet the inclusion criteria, but which were excluded, and explain why they were excluded.	6
Study characteristics	17	Cite each included study and present its characteristics.	6
Risk of bias in studies	18	Present assessments of risk of bias for each included study.	6
Results of individual studies	19	For all outcomes, present, for each study: (a) summary statistics for each group (where appropriate) and (b) an effect estimate and its precision (e.g. confidence/credible interval), ideally using structured tables or plots.	15-16
Results of syntheses	20a	For each synthesis, briefly summarize the characteristics and risk of bias among contributing studies.	6
	20b	Present results of all statistical syntheses conducted. If meta-analysis was done, present for each the summary estimate and its precision (e.g. confidence/credible interval) and measures of statistical heterogeneity. If comparing groups, describe the direction of the effect.	20-21
	20c	Present results of all investigations of possible causes of heterogeneity among study results.	7
	20d	Present results of all sensitivity analyses conducted to assess the robustness of the synthesized results.	7
Reporting biases	21	Present assessments of risk of bias due to missing results (arising from reporting biases) for each synthesis assessed.	7
Certainty of evidence	22	Present assessments of certainty (or confidence) in the body of evidence for each outcome assessed.	7
**DISCUSSION**			
Discussion	23a	Provide a general interpretation of the results in the context of other evidence.	9
	23b	Discuss any limitations of the evidence included in the review.	9
	23c	Discuss any limitations of the review processes used.	10
	23d	Discuss implications of the results for practice, policy, and future research.	10
**OTHER INFORMATION**			
Registration and protocol	24a	Provide registration information for the review, including register name and registration number, or state that the review was not registered.	10
	24b	Indicate where the review protocol can be accessed, or state that a protocol was not prepared.	10
	24c	Describe and explain any amendments to information provided at registration or in the protocol.	10
Support	25	Describe sources of financial or non-financial support for the review, and the role of the funders or sponsors in the review.	10

Competing interests	26	Declare any competing interests of review authors.	11
Availability of data, code and other materials	27	Report which of the following are publicly available and where they can be found: template data collection forms; data extracted from included studies; data used for all analyses; analytic code; any other materials used in the review.	11-14

Supplementary Figure S1. Funnel plots based on various key outcomes: A) HCV genotype. B) Liver cirrhosis, C) Decompensated liver cirrhosis, D) Sofosbuvir/ Velpatasvir-experienced HCV patients, and E) Active hepatocellular carcinoma

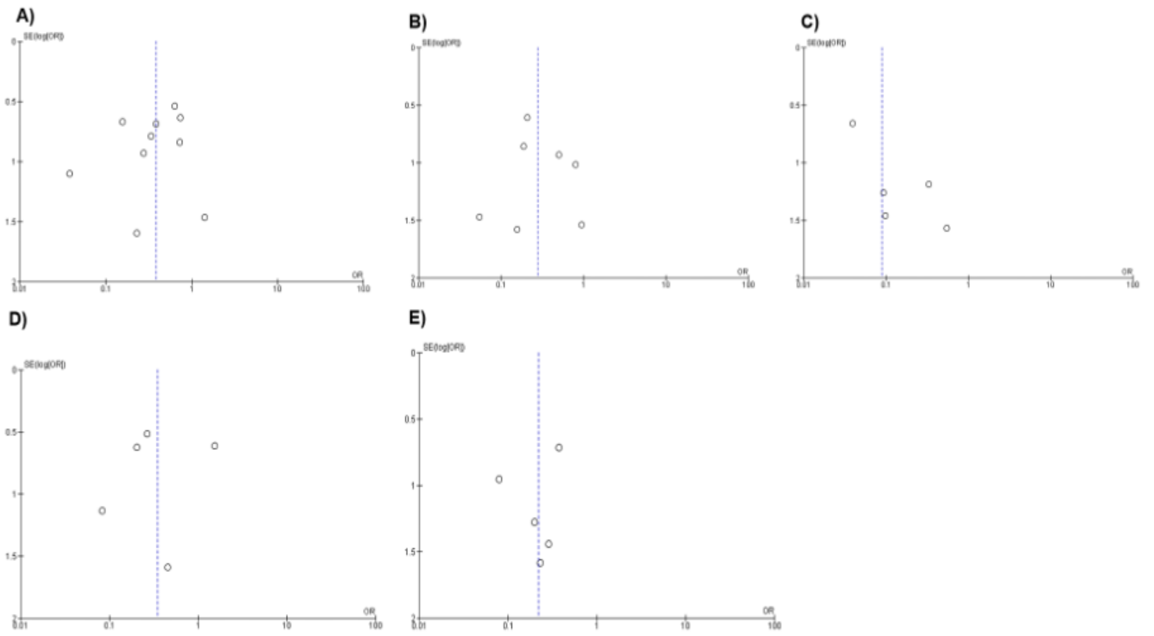

Supplementary Figure S2. SVR12 data based on WHO region classification.

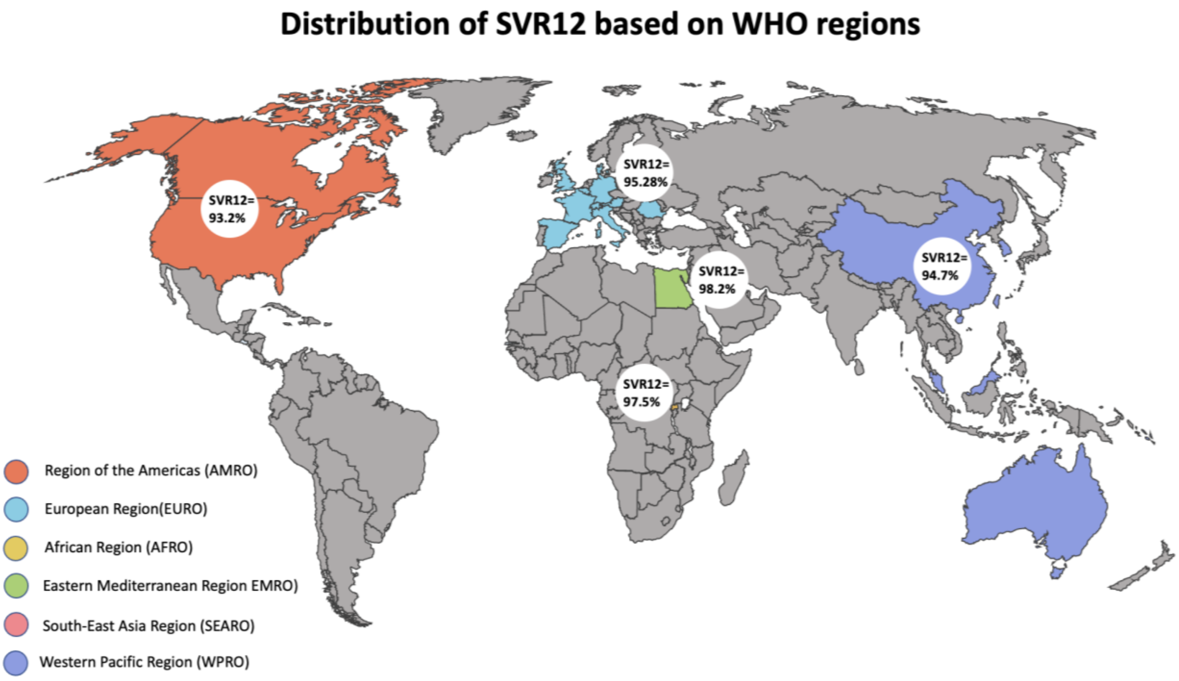

Supplementary Figure S3. The overall SVR12 rate of patients who had RAS mutation versus those with no RAS mutation

Study or Subgroup	RAS mutation Events	RAS mutation Total	No RAS Events	No RAS Total	Weight	Odds Ratio M-H, Random, 95% CI
Dagasperi 2019	86	88	19	20	41.6%	2.26 [0.20, 26.26]
Onofrio 2021	16	19	33	35	58.4%	0.32 [0.05, 2.13]
**Total (95% CI)**		**107**		**55**	**100.0%**	**0.73 [0.11, 4.77]**
Total events	102		52			

Heterogeneity: Tau² = 0.65; Chi² = 1.52, df = 1 (P = 0.22); I² = 34%
Test for overall effect: Z = 0.33 (P = 0.74)

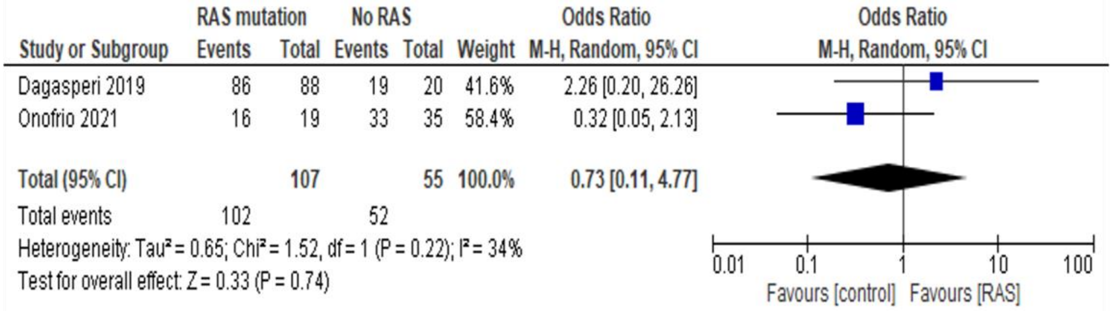

Supplementary Figure S4. The overall SVR12 rate of patients treated with Ribavirin versus without Ribavirin

Study or Subgroup	Ribavirin Events	Ribavirin Total	No RBV Events	No RBV Total	Weight	Odds Ratio M-H, Random, 95% CI
Dagasperi 2019	36	36	127	132	23.9%	3.15 [0.17, 58.29]
El-Kansas 2023	138	140	138	141	38.4%	1.50 [0.25, 9.12]
Papaluca 2021	23	26	100	102	37.7%	0.15 [0.02, 0.97]
**Total (95% CI)**		**202**		**375**	**100.0%**	**0.76 [0.12, 4.68]**
Total events	197		365			

Heterogeneity: Tau² = 1.40; Chi² = 4.41, df = 2 (P = 0.11); I² = 55%
Test for overall effect: Z = 0.30 (P = 0.76)

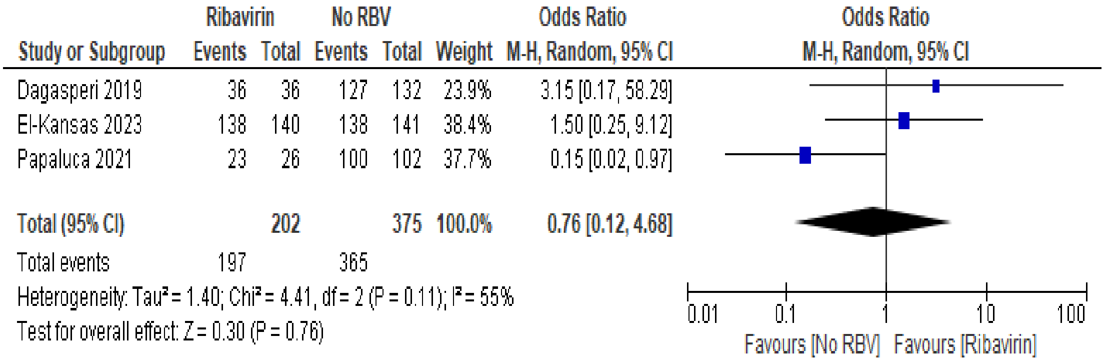

Supplement: Supplementary file 1 [file viruses-15-01489-s001.zip › viruses-2464964-supplementary materials_FINAL.pdf]
